# The Role of Discrimination, Childhood Maltreatment, and Social Determinants of Health in Adult BIPOC Pain Disparities

**DOI:** 10.1007/s40615-025-02422-9

**Published:** 2025-04-26

**Authors:** Andrea N. Trejo, Allan D. Tate, Amy E. Noser, Elizabeth Wieling, Alicia Kunin-Batson, Amanda Trofholz, Jerica M. Berge

**Affiliations:** 1 Department of Human Development and Family Science, East Carolina University College of Health and Human Performance, Greenville, NC USA; 2 Department of Epidemiology and Biostatistics, School of Public Health, University of Georgia, Athens, GA USA; 3 Department of Family Medicine & Community Health, University of Minnesota School of Medicine, Minneapolis, MN USA; 4 Department of Human Development and Family Science, School of Family and Consumer Sciences, University of Georgia, Athens, GA USA; 5 Department of Pediatrics, University of Minnesota School of Medicine, Minneapolis, MN USA; 6 Center for Learning Health System Sciences, University of Minnesota Medical School, Minneapolis, MN USA; 7 Department of Family Medicine and Adult and Child Center for Outcomes Research and Delivery Science (ACCORDS), University of Colorado Anschutz Medical Campus, Aurora, CO USA

**Keywords:** Chronic pain disparities, Social determinants of health (SDoH), Discrimination and pain, Childhood maltreatment, BIPOC health disparities, Trauma and chronic stress

## Abstract

**Objectives:**

Chronic pain disproportionately affects Black and Indigenous people and other people of color (BIPOC). Disparities may be related to increased chronic stress due to discrimination, trauma exposure, and social determinants of health (SDoH).

**Methods:**

Using data from families (*n* = 1307) in the family matters study (collected 2017–2019), a secondary data analysis explored SDoH of baseline pain severity and change in pain at 18 months, and the moderating effects of childhood maltreatment and discrimination on SDoH-pain relationships. General estimating equations (GEE) modeling was used.

**Results:**

Childhood maltreatment was associated with higher baseline pain severity, and discrimination was the strongest correlate of worse pain 18 months later. Childhood maltreatment exacerbated risk for higher pain severity for women, individuals under the federal poverty line, and individuals living in areas with low financial privilege. Discrimination increased risk for higher baseline pain for Black and Latinx individuals.

**Conclusions:**

Discrimination and traumatic event exposure may be important contributors to BIPOC pain disparities. Pain interventions may benefit from additional attention to the toll of oppressive systems and chronic stressors on BIPOC health.

Chronic pain, defined as pain persisting for at least three months, affects approximately one third of Americans [1]. Chronic pain is a significant reason for the pursuance of disability, costs the US government over $500 billion per year, and is associated with higher mortality rates and lower quality of life [1]. Black and Indigenous people, and other people of color (BIPOC) are disproportionately impacted by chronic pain. Disparities are commonly attributed to access barriers, such as insurance status and the ability to afford care. Although more scholars are considering the health impacts of structural racism, less attention is paid to the role of racialized chronic stressors (e.g., discrimination). The current study explored associations between social determinants of health (SDoH) and pain outcomes, and the moderating effects of childhood maltreatment and discrimination, in a racially and ethnically diverse adult sample located in the Twin Cities, Minnesota.

## Risk Factors for BIPOC Pain Disparities

SDoH are defined as the social, economic, and environmental factors that influence health outcomes, such as socioeconomic status, access to healthcare, and living conditions [2]. Structural oppression, such as racism and other forms of discrimination, plays a key role in shaping these determinants. Health disparities and healthcare access barriers among BIPOC have been extensively documented and supported throughout a range of settings, including pain medicine [3–5]. Black patients report worse pain severity, higher disability, more anxiety around pain, more activity avoidance, and higher depressive symptoms than their Euro-White counterparts [4, 5]. Black and Latinx chronic pain patients also receive lower quality pain management care across various conditions and healthcare settings [3, 4]. Although limited, research has also demonstrated similar pain disparities for Indigenous and multi-racial people [3].

More scholars have argued that racism drives racialized SDoH through two mechanisms [3, 6]. First, historical and ongoing racial stratification creates social, economic, environmental, and health inequities [3]. For example, BIPOC are at higher risk of poor healthcare access, lower quality care, living in polluted areas, and economic stress [6]. Additionally, BIPOC are more likely to be exposed traumatic events, including hate crimes, race-based violence, and childhood maltreatment, on top of historical and/or intergenerational trauma [3, 7, 8]. These disparities compound upon other social and biological factors, such as identifying as a woman and being assigned female at birth, both of which increase the likelihood of chronic pain and related conditions due to both gendered and biological influences [9].

Second, the chronic stress of living in a racially stratified society causes biological weathering (allostatic overload), directly linked with poor health outcomes, including chronic pain. The body is typically well-equipped to manage momentary stressors through a physiological process called allostasis [10]. A cascade of physiological responses is initiated to prepare the body to respond to threat, followed by processes to return the body to homeostasis once the threat has passed [10]. However, BIPOC are often exposed to stressors that do not have a clear starting or ending point and may continue throughout the lifespan. In addition, the emotional toll of traumatic events can be chronic. Allostasis becomes destructive when continuously activated for long periods of time, as is the case with chronic toxic stress. Chronic allostasis can result in the body building a tolerance to the amounts of chemicals required throughout a stress response; our tolerance point for activation and the shut-off response increases [10]. When experiencing chronic stressors without reprieve, allostasis also persists longer than intended without shutting off [10]. This process interrupts opportunities for homeostasis and recovery [10, 11]. This results in allostatic load, and eventually allostatic overload, or long-term damage due to chronic activation of the stress response [10, 11].

Chronic stress has been associated with various poor health outcomes [10, 12, 13]. There are multiple physiological pathways through which these physical consequences to long-term stress can occur, a major one being the link between allostatic overload and increased inflammation [14–16]. Chronic inflammation is one major contributor to pain and pain-related illnesses, such as arthritis and fibromyalgia [10, 11, 17–20]. Individuals from minoritized groups, including BIPOC, are more likely to experience a variety of chronic stressors across the lifespan as a direct result of racism and other oppressive ideologies. BIPOC are more likely to be exposed to traumatic events in childhood and adulthood due to structural inequities, hate crimes, and race-based violence, among other factors [21–26].

Discrimination is a form of chronic stress many BIPOC experience since childhood [2]. Discrimination has been associated with increased same-day cortisol, inflammation, and autoimmune activity, all of which are associated with pain [27, 28]. Discrimination also exacerbates risk for increased traumatic event exposure through various mechanisms, especially through violence motivated by racism, xenophobia, and other systems of oppression [29]. Additionally, several scholars argue that experiencing discrimination can be traumatic [30].

Childhood trauma may be another pathway through which allostatic overload may be kickstarted. BIPOC children are overrepresented in child abuse reports, especially Black, Native American/Indigenous, Pacific Islander, and multiracial children [8]. Aside from caseworker reporting bias, historical racial stratification of resources and opportunities may be a significant reason for these disparities [8, 31]. Low economic resources, housing instability, parental substance abuse and mental health difficulties, and parental history of childhood trauma and intimate partner violence are all risk factors for child maltreatment (the sexual, physical, emotional, verbal abuse, or neglect of a child) [8, 31–33].

Unfortunately, these risk factors thrive in conditions in which people are forced to focus all their energy and resources on survival. For example, systemic racism has created conditions in which BIPOC communities may have to prioritize economic survival above all else, including mental health. In addition to the contributions of systemic racism on economic disparities, housing discrimination, and disproportionate trauma exposure, communities with majority BIPOC residents are often located in mental health deserts or limited in appropriate resources. Even when someone *is* able to access mental health support, they still risk being met with provider discrimination, lack of interpreters and multilingual clinicians, and lack of widely accessible evidence-based, culturally responsive interventions [34, 35].

The impacts of childhood maltreatment are multi-generational. Traumatic stress has painful impacts on emotion regulation, cognitive development, social well-being, and parenting efficacy; it also increases the risk of developing other mental health conditions such as depression and substance abuse [36]. As previously stated, parents with histories of childhood abuse are more likely to perpetrate childhood abuse themselves; life stressors coupled with impaired emotion regulation may make effective parenting incredibly difficult to do while also managing traumatic stress [37]. Further, childhood adversity leaves a physical wound. Adverse childhood experiences have been linked to increased risk for various diseases, such as cardiovascular disease, lung disease, and cancer [38]. Traumatic stress, which can develop in response to childhood maltreatment, has also been linked to poor health outcomes, including increased pain and increased inflammation [39, 40].

## The Exacerbating Role of Chronic Stress

The disproportionate exposure of BIPOC to adverse SDoH may partly explain disparities in chronic pain. However, chronic stress may also be a significant contributor. Not only is chronic stress an independent risk factor for pain, but it may also amplify the effects of other risk factors. For example, individuals facing economic insecurity may have heightened nervous system dysregulation if they also endure chronic discrimination or have a history of childhood trauma. Ongoing toxic stress such as discrimination or trauma may compound socioecological risk factors, increasing susceptibility to chronic pain or worsening the pain experience. Given these overlapping stressors, the moderating role of discrimination and childhood maltreatment in the relationship between SDoH and pain outcomes should be examined.

## Current Study

Based on the prior literature, the current study proposes exposure to discrimination and traumatic childhood events are both forms of chronic, toxic stress disproportionately experienced by BIPOC, which may amplify overall risk for developing pain, exacerbate other contributing factors, and affect the maintenance of pain over time. Furthermore, we have not found any literature investigating the existence of pain change disparities—differences between groups in how pain improves, plateaus, or worsens over time—in BIPOC.

The current study explored the following research questions through a secondary analysis, utilizing data collected from a racially/ethnically and socioeconomically diverse adult sample living in Minneapolis/St. Paul, Minnesota:

1. What are the social determinants of self-reported pain severity and change in pain in a diverse adult sample living in Minneapolis/St. Paul followed over two time points (18 months apart)?
2. Does childhood maltreatment modify the relationship between SDoHs with pain severity and change in pain over 18 months?
3. Does perceived discrimination modify the relationship between social determinants with pain severity and change in pain over 18 months?

Based on these research questions, we hypothesize that:

1. Greater exposure to adverse SDoH (e.g., lower household income, higher income and racial neighborhood polarization, being underemployed, being uninsured), being assigned female at birth, and higher depressive symptoms will be associated with higher initial pain severity and persistently high or worsened pain at follow-up.
2. Childhood maltreatment will amplify the relationship between risk factors and pain outcomes.
3. Discrimination will also exacerbate the relationship between risk factors and pain outcomes.

This study aims to contribute to the literature around structural determinants of pain disparities across various racial and ethnic groups and clarify potential areas of intervention and prevention. Exploring the influence of childhood adversity and discrimination on the relationship between SDoH and pain in a diverse sample may supplement existing understandings of the harmful effects of chronic, structural stressors across various racial and ethnic groups.

## Method

Data was collected through the *Family Matters* study, a longitudinal, mixed-methods cohort study examining risk and protective factors for child whole-person health. The University of Minnesota’s Institutional Review Board approved the *Family Matters* study, and all participants consented in accordance with the Declaration of Helsinki procedures [41]. The parent study recruited the primary guardians of children ages 5–7 years who also identified as Black/African American, Latinx, Hmong, Somali/Ethiopian, Native American/Indigenous, or Euro-White and lived in the Twin Cities area in Minnesota. Recruitment was stratified by race and ethnic group to ensure representation of underrepresented racial and ethnic groups. Families were recruited from primary care settings. Families with children who had had a recent well-child visit were recruited via a letter from their primary care clinic. Adults participated in a baseline survey and a follow-up survey 18 months later. The current study consisted of a secondary data analysis of data collected from parents at baseline (*N* = 1307) and 18 months follow-up (*N* = 1152). The full sample was utilized for research questions pertaining to the pain severity outcome. The sample was restricted to participants who completed both baseline and 18-month follow-up in analyses using pain change as the outcome variable.

### Outcome Variables

Outcome variables included pain severity at baseline and pain change at 18 months. Pain severity at both waves were measured using an item from the 36-Item Short Form Health Survey, Version 2 (SF-36 v2) [42]. At baseline and 18 months follow-up, participants were asked, “How much bodily pain have you had in the past month?” Participants rated their physical pain in the past month on a 5-point Likert scale (1 = none, 6 = very severe). Pain ratings were used to indicate pain severity at time 1. Pain change was calculated using a change score by subtracting baseline reported pain severity from 18-month pain severity.

### Predictor Variables

All predictors were assessed at baseline and 18-month follow-up, apart from self-reported history of childhood maltreatment which was assessed only at baseline. Baseline predictor values were utilized in analyses for baseline pain severity, and 18-month follow-up predictor values were utilized in analyses for pain change.

#### Childhood Maltreatment

Exposure to childhood maltreatment was defined as a subset of adverse childhood experiences (ACEs) [43]. ACEs were measured at baseline, where participants reported experiences such as parental divorce, family member mental illness, family member imprisonment, food instability, lack of emotional support, parental substance abuse, physical abuse by parent, verbal abuse by parent, sexual abuse by parent, and sexual abuse by someone other than a parent.

Recent literature suggests separating ACEs items into maltreatment and deprivation (household dysfunction) [43–45]. Maltreatment items strongly predict mental health issues [43–45]. Following this, we isolated ACEs items related to maltreatment to create a childhood maltreatment variable. Using Negriff's approach [44] with modifications, we included six ACEs items: lack of emotional support, physical abuse, verbal abuse, sexual abuse by parent, sexual abuse by someone else, and witnessing parental violence. Deviations from Negriff’s maltreatment measure include excluding food insecurity, which may be linked to lack of resources rather than intentional neglect, and the inclusion of witnessing parental violence due to its similar impact on mental health [44].

Indicator-coded variables represented the presence of each adverse childhood experience. A mean of endorsed items was calculated and transformed into 10% units for interpretability. Cronbach’s alpha was 0.80, suggesting good internal consistency in this sample.

#### Discrimination

Frequency of experiencing discrimination in the past year was assessed at baseline and at 18-month follow-up using a measure adapted from Neumark-Sztainer and colleagues [46, 47]. Cronbach’s alpha was 0.83, suggesting good internal consistency for this measure in this sample. Parents were asked how often they have been “teased or harassed” about their race, religion, financial discrimination, their weight, and their appearance, and how often they had been teased or harassed in a sexual manner. Parents were also asked how often they had been treated with less respect or courtesy, and how often they had received poorer service or treatment than other people at restaurants, stores, doctor offices, or hospitals. Participants indicated frequency on a 5-point Likert scale (1 = never, 2 = less than once a year, 3 = a few times per year, 4 = a few times per month, 5 = at least once per week). The six items were operationalized as a mean score.

#### Depressive Symptoms

Participant depressive symptoms in the past month were assessed at baseline and 18-month follow-up using the Kessler Screening Scale for Psychological Distress (K6) [48]. Participants reported frequency of experiencing the following symptoms in the past month: hopelessness, nervousness, feeling restless or fidgety, feeling “so depressed nothing could cheer you up,” feeling “everything was an effort,” and worthlessness. Responses were rated on a 5-point Likert scale, in which 1 = all of the time, 2 = most of the time, 3 = some of the time, 4 = a little of the time, and 5 = none of the time. Items were reverse scored, summed, and averaged. Cronbach’s alpha was 0.92 indicating strong reliability for this measure in this sample.

### Social Determinants of Health

The following SDoH variables were also included to address structural influences on health: level of formal education, insurance status, income, employment status, census-tract income inequity, and nativity status.

#### Level of Formal Education

Participants reported their highest level of formal education at baseline (some high school, high school, some college, Bachelor’s degree, and graduate degree).

#### Insurance Status

Participants reported their insurance status and insurance coverage type. Coverage was then binarized into uninsured and insured.

#### Income

Annual household income level was reported at baseline (< $20,000, $20,000–$34,000, $35,000–$49,000, $50,000–$74,900, and $75,000 +).

#### Employment

Participants reported their current work status (full-time, part-time, stay-at-home caregiver, currently unemployed but actively seeking work, and not working for pay).

#### Nativity Status

Participants indicated whether they were born inside or outside the USA.

#### Census Tract Income Inequity

Census tract income inequity was defined by the distribution of income at the census tract level and is measured using the Index of Concentration at the Extremes [49]. Census tract income inequity was rated from 1 to 5 each representing quintiles of census tracts, with a score of 1 indicating high homogeneity of low income (e.g., concentrated poverty in the bottom 20% of tracts) and 5 indicating high homogenous high income (e.g., concentrated economic privilege in the upper 20% of sample tracts). The central value of 3 indicates not concentrated high or low income at the tract level.

### Study Demographics and Covariates

Sex, age, and race/ethnicity were included to control for demographic characteristics and depressive symptoms.

#### Sex

Participants were asked to report their sex at baseline as either male or female.

#### Age

Participant age was operationalized at baseline, using self-reported date of birth.

#### Race and/or Ethnicity

Participants identified as Euro-White, Black and/or African American, Latino/a/e, Hmong, Somali and/or Ethiopian, and Native American (Table 1).

**Table 1:** Participant demographics and descriptives

	*N* (%)	Mean (SD)	Min	Max
Women	1172 (89.67)			
Age		35.67 (7.91)	19.85	73.09
Race and ethnicity				
White	239 (18.29)			
Black	280 (21.42)			
Latino/a/e	215 (16.45)			
Hmong	226 (17.29)			
Somali/Ethiopian	136 (10.41)			
Native American	211 (16.14)			
Highest level of formal education				
Less than high school	211 (16.14)			
High school or GED	322 (24.64)			
Some college	421 (32.21)			
Bachelor’s degree	187 (14.31)			
Graduate or professional degree	166 (12.70)			
Born in the USA	860 (65.80)			
Insurance status				
Uninsured	155 (11.86)			
Insured	1152 (88.14)			
Employment status				
Full-time	588 (44.99)			
Part-time	276 (21.12)			
Stay-at-home caregiver	203 (15.53)			
Unemployed, seeking employment	121 (9.26)			
Not working for pay	119 (9.10)			
Household income				
Less than $20,000	393 (30.32)			
$20,000–$34,000	323 (24.92)			
$35,000–$49,000	203 (15.66)			
$50,000–$74,900	143 (11.03)			
$75,000 +	234 (18.06)			
Childhood maltreatment		1.30 (2.25)	0	10
Discrimination		2.22 (0.40)	1	4.63
Depressive symptom severity		1.75 (0.86)	1	5

Total *N* = 1307*.* Data was collected in the Twin Cities, MN

### Statistical Analysis

Analyses were performed in Stata 17MP (College Station, TX). Pairwise comparison of estimated marginal means was used to determine whether between-racial group differences in pain severity and change in pain were present in our sample. Generalized estimating equations (GEEs) were used to estimate a marginal model for the main effects of SDoH on baseline pain severity. Multiple linear regressions were used to test the main effects of SDoH on pain change at 18 months. A GEE population-averaged model, using a Gaussian family, identity link, independence working correlation structure, and Huber-White robust standard errors, was used to estimate average marginal effects for interactions between childhood maltreatment and SDoH, and discrimination and SDoH, on pain [50–52]. GEE was used to account for a repeated measures design. All interaction terms were fitted in separate models, and multiplicity adjustment was not conducted due to the family of prespecified analyses and our presentation of all null findings in line with prior expert guidance [53]. All analyses controlled for sex, age, and race/ethnicity as well as baseline education, insurance status, income, employment status, census tract income inequity, nativity status, and depressive symptoms.

## Results

The sample size of 1307 adults had a mean age of 35.67 years; the youngest participant was 19.85 years old and the oldest was 73.09 years. Most participants were assigned female sex at birth (89.67%) and were born in the USA (65.8%). Around 21% (21.42%) of participants were Black, 18.29% White, 17.29% Hmong, 16.45% Latinx, 16.14% Native American/Indigenous, and 10.41% Somali/Ethiopian. Eighty-eight percent of participants were insured, and almost 45% reported working full-time. Over half of participants (55.24%) had a household income under $34,001. Almost 35% of participants reported history of childhood maltreatment, although the number of events reported was low (*M* = 1.31, *SD* = 2.3*,* range = 0–10). On average, depressive symptoms were low (*M* = 1.75, *SD* = 0.86) and reported discrimination (*M* = 2.22, *SD* = 0.4) was moderately low.

Baseline and follow-up mean pain ranged from “very mild” to “mild” on average. Pain severity also remained relatively stable on average between assessments, with a small downward trend (*M* = − 0.04, *SD* = 1.3). Exploratory analyses were conducted to examine differences in pain experiences between participants of various racial and/or ethnic groups in our sample. Black and Native American participants reported higher baseline pain severity than other racial/ethnic groups. Of participants who reported pain onset at follow-up, 25% identified as White, 21.15% as Black, and 19.23% as Hmong.

### Social Determinants of Pain Severity and Pain Change

Increased exposure to childhood maltreatment, discrimination, and depressive symptoms were associated with higher baseline pain. Income between $50,000 and $74,900 was associated with lower baseline pain. Discrepancies in initial pain severity existed between Indigenous participants and participants of other racial/ethnic groups except Latinx participants. Discrimination was the only predictor associated with higher pain at 18 months. See Table 2 for results.

**Table 2:** Social determinants of pain level and pain change

	Baseline pain severity	C.I	Pain change	C.I
Childhood maltreatment	**0.08**	**[0.05,0.12]**	− 0.02	[− 0.06,0.02]
Discrimination	**0.15**	**[0.03,0.27]**	**0.33**	**[0.16,0.51]**
Sex (1 = woman)	0.11	[− 0.06,0.29]	0.18	[− 0.07,0.42]
Age	**0.02**	**[0.01,0.03]**	0.01	[− 0.01,0.02]
Race and ethnicity				
White (reference group)				
Black	0.09	[− 0.14,0.32]	0.00	[− 0.29,0.29]
Latino/a/e	0.12	[− 0.16,0.40]	0.09	[− 0.27,0.44]
Hmong	− 0.05	[− 0.28,0.18]	0.08	[− 0.22,0.38]
Somali/Ethiopian	− 0.10	[− 0.42,0.22]	0.26	[− 0.13,0.65]
Native American	**0.33**	**[0.07,0.58]**	− 0.08	[− 0.38,0.21]
Formal education				
Some high school (reference group)				
High school	0.18	[− 0.01,0.37]	− 0.10	[− 0.38,0.18]
Some college	0.16	[− 0.03,0.34]	− **0.33**	**[**− **0.61,** − **0.05]**
Bachelor’s degree	0.09	[− 0.14,0.32]	− 0.21	[− 0.52,0.10]
Graduate school	− 0.01	[− 0.26,0.24]	− 0.24	[− 0.58,0.10]
Nativity status (1 = US born)	0.08	[− 0.10,0.25]	0.06	[− 0.19,0.31]
Insurance status (1 = uninsured)	− 0.04	[− 0.22,0.15]	0.16	[− 0.14,0.45]
Employment status				
Full-time (reference group)				
Part-time	0.07	[− 0.07,0.21]	− 0.02	[− 0.22,0.18]
Stay-at-home caregiver	− 0.05	[− 0.22,0.13]	− 0.10	[− 0.37,0.16]
Unemployed, seeking employment	0.14	[− 0.06,0.34]	0.05	[− 0.26,0.36]
Work for no pay	0.17	[− 0.08,0.41]	− 0.06	[− 0.40,0.29]
Household income				
Less than $20,000 (reference group)				
$20,000–$34,000	− 0.15	[− 0.31,0.01]	0.00	[− 0.25,0.25]
$35,000–$49,000	− 0.15	[− 0.34,0.04]	0.03	[− 0.25,0.30]
$50,000–$74,900	− **0.28**	**[**− **0.49,** − **0.07]**	0.15	[− 0.16,0.46]
$75,000 +	− 0.20	[− 0.45,0.05]	0.27	[− 0.06,0.59]
Census tract income inequity				
1 (reference group)				
2	0.11	[− 0.06,0.28]	0.06	[− 0.17,0.30]
3	0.02	[− 0.16,0.20]	− 0.03	[− 0.31,0.26]
4	0.10	[− 0.08,0.27]	0.01	[− 0.22,0.24]
5	0.18	[− 0.02,0.38]	− 0.03	[− 0.27,0.21]
Depressive symptoms	**0.41**	**[0.32,0.49]**	0.09	[− 0.01,0.20]
Constant	0.06	[− 0.51,0.64]	− 1.34	[− 2.17, − 0.50]

*N* = 1307. Values presented are betas. Census Tract Income Inequity, 1 = high homogenous low income, 5 = high homogenous high income. Data was collected in the Twin Cities, MN

### Moderating Effects

Childhood maltreatment interacted with sex, income, and neighborhood income inequity on baseline pain severity (see Table 3). Childhood maltreatment amplified risk for higher baseline pain for female participants; a unit increase in childhood maltreatment was associated with a 0.09 unit increase in pain severity at baseline [0.06, 0.13]. Childhood maltreatment also exacerbated the relationship between low income and higher baseline pain for individuals under the federal poverty line, which provided some evidence of a threshold effect. Finally, childhood maltreatment had unfavorable synergistic effects on elevated baseline pain in census tracts with more extreme economic polarization. Childhood maltreatment was also associated with lower pain at 18 months for individuals living in the first income bracket under the federal poverty line. The pattern of modification may not be clear across the income distribution. A moderating effect of discrimination on the relationships two racial/ethnic groups and pain outcomes was supported. Black and Latinx individuals appeared to be more susceptible to the negative effects of discrimination on baseline pain than other racial/ethnic groups. See Table 4.

**Table 3:** Moderating effects of childhood maltreatment

	Baseline pain level							Pain change						
Interaction	Strata marginal effects	Lower C.I	Upper C.I	*SE*	Chi-square	df	Sig	Strata marginal effects	Lower C.I	Upper C.I	*SE*	Chi-square	df	Sig
1. Maltreatment × sex					13.6	1	0.00***							
Male	− **0.07**	− 0.14	0.00	0.04										
Female	**0.09**	0.06	0.13	0.02										
5. Maltreatment × income					12.0	4	.02*					10.0	4	0.04*
Less than $20,000	**0.12**	**0.07**	**0.17**	**0.03**				− 0.03	− 0.11	0.05	0.04			
$20,000–$34,000	**0.11**	**0.05**	**0.16**	**0.03**				− **0.09**	− **0.16**	− **0.01**	**0.04**			
$35,000–$49,000	0.03	− 0.04	0.10	0.03				0.01	− 0.08	0.11	0.05			
$50,000–$74,900	0.01	− 0.05	0.07	0.03				**0.07**	**0.00**	**0.15**	**0.04**			
$75,000 +	0.04	− 0.03	0.10	0.03				− 0.01	− 0.07	0.05	0.03			
7. Maltreatment × census-tract income inequity					19.0	4	0.00***							
High low-income homogeneity: 1	**0.13**	**0.08**	**0.17**	**0.02**										
2	0.00	− 0.07	0.08	0.04										
3	0.00	− 0.07	0.07	0.03										
4	**0.14**	**0.07**	**0.21**	**0.03**										
High high-income homogeneity: 5	0.02	− 0.05	0.09	0.04										

The strata marginal effect coefficient indicates the change in the pain outcome per one standard deviation increase in discrimination. Significant results are bolded. *C.I.* confidence interval. *p < 0.05; **p < 0.010; ***p < 0.001***. Data was collected in the Twin Cities, MN

**Table 4:** Moderating effects of discrimination, outcome: pain severity

Interaction	Strata marginal effects	Lower C.I	Upper C.I	*SE*	Chi-square	df	Sig
Discrimination × race/ethnicity					11.58	5	0.04*
White	− 0.1	− 0.35	0.15	0.13			
Black	**0.3**	**0.02**	**0.59**	**0.14**			
Latino/a/e	**0.3**	**0.13**	**0.49**	**0.09**			
Hmong	0.1	− 0.23	0.45	0.17			
Somali/Ethiopian	0.2	− 0.19	0.66	0.22			
Native American	− 0.1	− 0.38	0.17	0.14			

The strata marginal effect coefficient indicates the change in the pain outcome per one standard deviation increase in discrimination. Significant results are bolded. No significant moderating effects were found for pain change by discrimination. *C.I.* confidence interval. *p < 0.05; **p < 0.010; ***p < 0.001. Data was collected in the Twin Cities, MN

## Discussion

Childhood maltreatment and recent discrimination may contribute to the severity and change in pain. Childhood maltreatment emerged as the most significant predictor for elevated pain severity at baseline, while experiencing discrimination in the last month was the strongest correlate of worsened pain at 18 months. Childhood maltreatment exacerbated baseline pain severity for groups with less systemic privilege, such as women, individuals under the federal poverty line, and individuals living in under-resourced areas. Discrimination amplified the risk for elevated baseline pain for Black and Latinx individuals. Identifying as Native American/Indigenous remained associated with higher baseline pain, which suggests the presence of additional factors exacerbating risk not included in our model. Further research would be useful in understanding additional risk factors present for Indigenous populations with pain.

These results may support the theory of compounding chronic stressors (e.g., trauma, financial stress) exacerbating pain outcomes. Exploring the socioeconomic distribution of participants according to racial/ethnic group reveals notable trends. Almost half of White participants (*M* = 0.49) lived in census-tracts with the highest economic privilege, versus Black (*M* = 0.13), Latinx (*M* = 0.13), Hmong (*M* = 0.05), Somali/Ethiopian (*M* = 0.11), and Native American participants (*M* = 0.09). White participants also had the lowest percentage of residence (*M* = 0.03) in low-resourced areas, compared to Black (*M* = 0.24), Latinx (*M* = 0.16), Hmong (*M* = 0.19), Somali/Ethiopian (*M* = 0.14), and Native American participants (*M* = 0.25). These realities support existing empirical evidence of racialized income inequity, which places communities at further risk for experiencing chronic stress.

### Public Health Implications

Interventions aimed at supporting BIPOC communities in healthcare should address the health impacts of oppressive societal structures, while providing tools and strategies to foster health through this lens. Improving the triage process of pain patients affected by childhood maltreatment and/or traumatic stress may be beneficial to trauma-affected chronic pain patients. The significant impact of childhood maltreatment in amplifying risk factors and contributing to pain incidence highlights the importance of increasing access to evidence-based traumatic stress interventions for those with childhood trauma exposure. Supporting individuals affected by traumatic stress—a form of chronic stress—may not only decrease pain risk but also improve their ability to access resources and engage in pain prevention and management strategies.

Screening for traumatic exposures and symptoms, followed by referral to evidence-based treatments for Post-Traumatic Stress Disorder, should be integrated into standard pain care. These efforts should be complemented by culturally responsive, anti-racist providers as a critical component of BIPOC mental healthcare, including behavioral pain management. Findings also highlight limited economic resources as a key determinant of pain. Financial resources serve as a central mechanism through which structural racism operates. Families who are under-resourced face significant barriers to healthcare access and experience heightened stress levels due to economic instability. Unfortunately, we cannot out-treat economic stress and insecurity. Without addressing these structural inequities, micro-level interventions may have limited effectiveness.

Additionally, the standard of care for pain management must be re-examined. At best, pain interventions and treatment plans often lack the adaptation and contextualization needed to fully support BIPOC pain patients. Beyond adaptation, scholars have called for the development of interdisciplinary, multimodal interventions that account for the complexity of pain management [54]. Treatment for chronic pain frequently requires coordination across multiple specialties, attention to various lifestyle factors, and multi-illness management. A holistic, integrated approach to pain management may ensure more comprehensive care while minimizing the burden on patients’ resources. For BIPOC communities, in particular, interventions must address the compounded effects of systemic inequities on pain and other health outcomes, necessitating a collaborative, interdisciplinary framework.

These findings contribute to a growing body of literature demonstrating the significant health consequences of systemic racism. Systemic racism, whether manifested through health disparities, economic inequities, or targeted violence, has measurable and devastating effects on BIPOC communities [29]. Effectively addressing these issues will require radical institutional reform. Such transformation should include reshaping American healthcare policy (e.g., implementing universal healthcare and expanding access to disability benefits), investing in healthcare-centered reparations to descendants of formerly enslaved Black people (e.g., paid healthcare, healthcare policy created *with* the Black community), and allocating government funding towards medical research led by and for BIPOC communities [55–58].

### Limitations

Recruitment was conducted in primary care settings and did not seek out chronic pain patients, though they are frequently identified and treated in primary care [59]. Further, our pain measure was limited to a single item, which constrains inferences regarding the clinical significance of reported pain severity and changes. While results should be interpreted with caution, this study offers an opportunity to begin exploring pain experiences within a uniquely racially and ethnically diverse, longitudinal sample. Future research incorporating additional data collection time points would strengthen the analysis of pain trajectories in this sample.

Additionally, our discrimination measure captured multiple forms of discrimination, rather than being limited to racial/ethnic discrimination. Most of the sample was female, which may limit the generalizability of findings to men's experience of pain. Unfortunately, we could not determine how many participants may have been transgender, nonbinary, or intersex, as the dataset only included binary options (male or female) for sex and did not include a separate measure for gender. Future pain research should explicitly incorporate participants of all genders and sexes to ensure more representative findings. Another limitation for this study was the assessment window for depressive symptoms, which spanned the past month—the same timeframe used for measuring pain severity. As a result, inferences regarding the depression-pain relationship are restricted. Further replication of these findings will be needed in other longitudinal studies to increase confidence that detected associations represent mechanisms of pain severity and change over time.

## Future Directions

Inclusion of biomarkers and more comprehensive pain measures represents an important next step in examining the role of discrimination and traumatic stress in perpetuating pain disparities. In future analyses, we hope to include cortisol and inflammatory markers to better understand the physiological pathway between chronic stress and pain. Additionally, future studies would benefit from incorporating traumatic stress measures to more precisely assess its impact on pain. Because this study used childhood maltreatment as a proxy for traumatic stress, our results are limited to the effects of traumatic event exposure rather than the broader impact of chronic traumatic stress. Further, employing a discrimination measure that specifically captures racial discrimination would provide deeper insight into the unique impact of racist discriminatory experiences on pain.

This study underscores the compounding effects of discrimination and childhood trauma on BIPOC pain experiences, as these adversities further intensify the effects of SDoH on pain. More broadly, this study highlights the unequal distribution of privilege and risk across racial/ethnic groups. By documenting one manifestation of structural racism’s toll on BIPOC health, we emphasize the urgency of developing targeted prevention and intervention strategies that specifically address BIPOC health amidst systemically-imposed oppression. Through structural reform and interdisciplinary collaboration, BIPOC patients may gain improved access to equitable, effective care and greater opportunities for wellness.

## Data Availability

Data is available upon request from the Principal Investigator (Berge).
